# Structure predictions of MuRF1‐UBE2 complexes identify amino acid residues governing interaction selectivity for each MuRF1‐E2 pair

**DOI:** 10.1111/febs.70017

**Published:** 2025-02-10

**Authors:** Agnès Claustre, Mélodie Malige, Maëlys Macheton, Lydie Combaret, Etienne Lefai, Pierre Fafournoux, Daniel Taillandier, Julien Henri, Cécile Polge

**Affiliations:** ^1^ Université Clermont Auvergne, INRAE, UNH Clermont‐Ferrand France; ^2^ Sorbonne Université, CNRS, Laboratoire de Biologie Computationnelle et Quantitative, Institut de Biologie Paris‐Seine, UMR 7238 Paris France

**Keywords:** MuRF1/TRIM63, protein–protein interaction selectivity, RING‐type E3 ligase, UBE2, ubiquitin

## Abstract

The RING‐type E3 ubiquitin–protein ligase MuRF1 (also known as TRIM63) plays an important role in skeletal muscle atrophy by targeting contractile proteins. *In cellulo*, MuRF1 can alternatively interact with four E2 enzymes (UBE2E1, UBE2J1, UBE2J2, or UBE2L3), suggesting different functions or targets for the four MuRF1‐E2 complexes. In this article, we studied the interface of these MuRF1‐UBE2 complexes based on AlphaFold2 and AlphaFold3 predictions. These predictions revealed the involvement of different residues at the interface of each complex. We confirmed this overall interface difference by the differential sensitivity of MuRF1‐E2 complexes to regenerating solutions in surface plasmon resonance experiments. We further confirmed several predictions individually by affinity measurements with point‐mutant E2 enzymes and truncated MuRF1. We used the interaction‐induced fluorescence change approach with fluorescent MuRF1. Besides canonical E2‐RING‐type E3 interactions, we were able to identify selective contact points between MuRF1 and its UBE2 partners. Furthermore, in the case of the MuRF1‐E2E1 pair, unlike the other MuRF1‐E2 pairs, the interaction may also be governed by a domain outside the RING domain. Since the function of RING‐type E3s is regulated by E2 enzymes, deciphering the mechanisms of selective recruitment of E2s by MuRF1 paves the way for the development of targeted therapeutics to fight muscle atrophy.

## Graphical abstract

MuRF1 is a major regulator of muscle atrophy, a side effect of chronic pathologies. As a RING‐type E3, MuRF1 depends on E2 enzymes to synthesize ubiquitin chains on substrates. MuRF1 can interact with four E2 enzymes, suggesting different physiological functions depending on the MuRF1‐E2 pair. The authors identified mechanisms underlying the recognition selectivity between MuRF1 and E2s, thus opening the way to the development of targeted therapeutics against muscle atrophy.

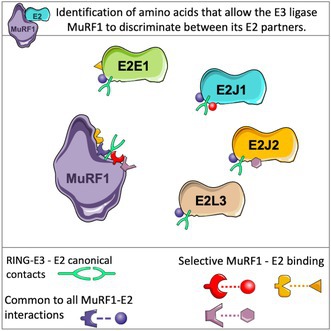

**Abbreviations**
AA: amino acid
AF: AlphaFold
E2: UBE2 enzyme
MFC: a MuRF‐specific domain
MuRF1_FL_
: MuRF1 full length
MuRF1_RM_
: corresponds to the first 115 amino acids of MuRF1 and includes the RING domain and the MuRF‐specific (MFC) domain
pLDDT: predicted local distance difference test
PPI: protein–protein interaction
RMSD: root mean square deviation
SPR: surface plasmon resonance
TRIM: tripartite motif E3 ligase
Ub: ubiquitin
UBC: ubiquitin‐conjugating catalytic
UPS: the ubiquitin–proteasome–proteolytic system
WT: wild‐type
To name the amino acids: we have used the three‐letter International Amino Acid Code

## Introduction

Several diseases (cancer, sepsis, congestive heart failure, kidney disease, etc.) are characterized by a catabolic state with massive muscle wasting. Consistently, low muscle mass in patients is a strong predictor of morbidity and mortality. Other outcomes reported to be associated with low muscle mass include reduced physical activity, lower quality of life, surgical complications, and severe treatment toxicity [1]. Muscle wasting is therefore a major public health issue. It is now recognized that the main determinant of muscle wasting in severe atrophic situations is increased skeletal muscle proteolysis, particularly of the most abundant proteins in muscle, the myofibrillar proteins. Among them, myofibrillar alpha‐actin and myosins are degraded by the ubiquitin–proteasome–proteolytic system (UPS) [2, 3, 4]. The UPS is thus a key player in the muscle atrophy process, making it a potential target for fighting the increased proteolysis that occurs during catabolic conditions.

Substrates degraded by the 26S proteasome are first labeled by a polyubiquitin (Ub) chain, a highly regulated process. The process is initiated by one of the two E1 ubiquitin‐activating enzymes that activates Ub and transfers it to the cysteine active site of an E2 Ub‐conjugating enzyme (38 members in humans). E3 Ub ligases (> 600 members predicted in the human genome) recognize the substrate to be degraded. In the case of RING‐type E3 ligases (90% of E3s), E2s provide catalytic activity, and their interaction with an E3 enzyme triggers the Ub transfer to the substrate [5, 6]. Depending on the E2, a specific Ub signal is built on the substrate, leading to specific substrate properties and fate [7]. One E2 can interact with multiple E3s and *vice versa*; it is thus the combination of E2 and E3 that confers the targeting specificity of the UPS. Thus, specific combinations of E2 and E3 enzymes are crucial for the control of muscle mass and represent potential clues for the development of new strategies against atrophy. However, this aspect of the UPS is underexplored, especially in skeletal muscle.

In the context of muscle atrophy, the RING‐type E3 ligase MuRF1, also known as TRIM63, is of particular interest and has been the focus of numerous studies. Indeed, this muscle‐specific E3 ligase is overexpressed in all catabolic conditions [8, 9, 10] and its deletion in mice protects the muscle from atrophy upon catabolic conditions [8, 11, 12, 13]. Furthermore, MuRF1 is the only E3 ligase known so far to target the contractile proteins (myofibrillar α‐actin and myosins, troponins, etc.) for degradation by the 26S proteasome [2, 14, 15, 16, 17]. This makes MuRF1 an attractive target for drug development. Potential strategies for preserving substrate degradation include (a) impeding substrate recognition [18, 19, 20, 21], (b) targeting the catalytic Cys of the E2, which may be largely harmful to the cell, (c) modulating the oligomeric state of the E3 [22], (d) interfering with Ub transfer onto the substrate [23], and (e) targeting specific regulatory surfaces like E2‐E3 interfaces. Our hypothesis is that drugs targeting specific E2‐MuRF1 interactions may be less harmful to muscle cells because such inhibition specifically inhibits one MuRF1‐E2 pair, that is, the pair involved in sarcomeric protein degradation.

E2 enzymes are characterized by an evolutionarily conserved catalytic core, the ubiquitin‐conjugating catalytic (UBC) fold of approximately 150 residues, including a catalytic cysteine that forms a covalent thioester bond with the C‐terminus of Ub. The UBC fold provides a binding platform for E1s and E3s [24]. E3s bind to α‐helix1 (α1) and loops L4 and L7 within the UBC domain. The interaction zone with the E1 enzyme overlaps the interaction zone with the E3 enzyme (helix H1) [6]. In addition to the UBC domain, some E2s contain an N‐terminal and/or C‐terminal extension, probably providing additional regulatory function(s). In RING‐type E3 ligases, the RING domain mediates the interaction with E2 [25]. The UBC domains of E2 show a high degree of structural conservation, as do the RING domains of E3. This is particularly true at the E2‐E3 interface, suggesting that a given E3 could potentially interact with all E2 enzymes and *vice versa*. However, a selectivity in E2‐E3 pairs formation is observed experimentally, with some pairs being exclusive [26, 27, 28]. How this selectivity between a dedicated E3 and its cognate E2 is achieved remains unclear. The identification of the molecular determinants underlying this selectivity could be a step toward the development of targeted therapeutics.

The published E3‐E2 complex structures display a well‐conserved hydrophobic interaction surface, including residues within the N‐terminus helix H1 and loops L4 and L7 of E2. In the case of RING‐type E3, these two loops contact the RING domain of E3 via a shallow groove containing an α‐helix and the two zinc chelator loops of the RING domain [25, 29, 30, 31, 32, 33]. However, despite the involvement of conserved elements at the E2‐E3 interface, some examples of E3‐E2 complexes revealed that specific residues are responsible for the interaction selectivity between an E2 and an E3 [31, 34, 35, 36]. For example, E2E1 and E2E2 interact with different E3 ligases, while the binding region of these E2s differs by only three residues [37]. On E2E2, the aspartate residue at position 59 (Asp59) allows specific binding to the E3 ligases Wwp1, Rbx2, and Ube3a. On E2E1, the residue at the same position, Glu105, plays a role in the interaction with Mdm2. Mutation of these residues destabilizes the MuRF1‐E2Es interactions [37].

A major difficulty of TRIM E3 studies is that, to our knowledge, none of them have been completely solved structurally, except when using deletions or point mutants [38]. Regarding MuRF1, the RING domain (E2 binding site) is still not solved. To gain further insight into the mechanism of MuRF1‐E2‐binding selectivity, we attempted to crystallize MuRF1‐E2 couples and to perform *in silico* predictions of the MuRF1‐E2 interfaces, using AlphaFold2 (AF2) and AlphaFold3 (AF3) predictions in the ColabFold setup or on the dedicated webserver, respectively [39, 40, 41]. We focused on four E2s expressed in muscle and interacting *in cellulo* with MuRF1, namely, UBE2E1, UBE2J1, UBE2J2, and UBE2L3 that we previously identified [42]. Some protein–protein consensual interface predictions made by both AF2 and AF3 were experimentally confirmed *in vitro*, and supported the involvement of a conserved hydrophobic interface, as previously observed in different E3‐E2 pairs. More interestingly, structural alignment of the four MuRF1‐E2 complexes highlighted the presence of both common and differential amino acid residues allowing the selectivity of interaction between MuRF1 and the various E2s.

## Results

### Structure predictions of the different MuRF1‐E2 pairs

**Fig. 1:**
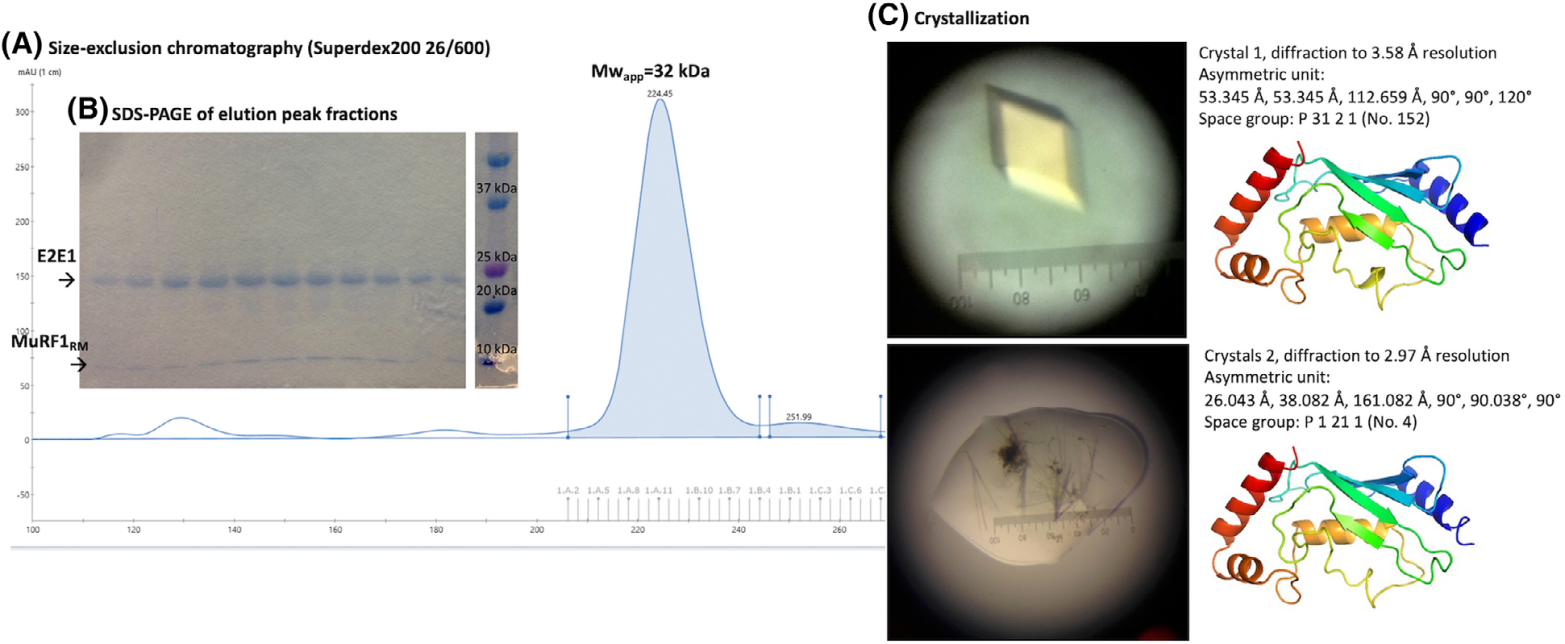
MuRF1_RM_‐E2E1 complex crystallization assay. (A) After affinity purification, recombinant MuRF1_RM_ and E2E1 were mixed at a 2 : 1 ratio and submitted to size exclusion chromatography (Superdex 200 26/600) to purify only the complex. (B) Fractions corresponding to the size of the dimer were analyzed by SDS/PAGE to confirm the presence of both MuRF1 and E2E1. (C) Purified MuRF1_RM_‐E2 complexes were submitted to extensive sparse‐matrix crystallization screens (384 JCSG conditions). Two conditions led to crystal formation. However, the two resolved structures represent E2E1 alone and the structure of E2E1 alone is already documented in the PDB (notably in entry code PDB: 3BZH). Protein purification to obtain the crystals was done once (*n* = 1). All structures and models are represented with pymol.

We first attempted to crystallize a portion of MuRF1, called MuRF1_RM_, with UBE2E1, UBE2L3, the cytosolic part of UBE2J1, and the cytosolic part of UBE2J2 that we previously identified as MuRF1 partners. MuRF1_RM_ corresponds to the first 115 amino acids (AA) of MuRF1 and includes the RING domain, which is the domain that interacts with the E2s, and the MuRF‐specific (MFC) domain. Despite standard sparse‐matrix screening over 384 conditions from the JCSG series, we only obtained crystals with reconstituted MuRF1_RM_‐E2E1 complex that later appeared to contain E2E1 alone (Fig. 1). We therefore decided to use *in silico* approaches. For structural predictions, we also used the sequence of MuRF1_RM_ and the full length of UBE2 enzymes. For simplification, UBE2 enzyme names will be abbreviated to E2 throughout the article, for example, the enzyme UBE2E1 will be referred to as E2E1.

AF2 and AF3 were used to predict the structure of the heterodimeric state of MuRF1_RM_ in complex with the E2 ubiquitin‐conjugating enzymes E2E1, E2J1, E2J2, and E2L3. Coverage in multiple sequence alignments ranges from 1000 to 2000 for MuRF1_RM_ to 4000 for E2L3, 5000 for E2J1 and E2J2, and 7000 for E2E1. Confidence metrics pLDDT is higher than 80 (reliable) for all but some local disordered sequences at residues 55–65 of MuRF1_RM_ and the amino‐termini and carboxy‐termini of individual domains. All three independently predicted complexes align with each other with reliable RMSD (< 2.3 Å) (Fig. 1), suggesting consistent computational modeling of protein–protein interaction (PPI).

**Fig. 2:**
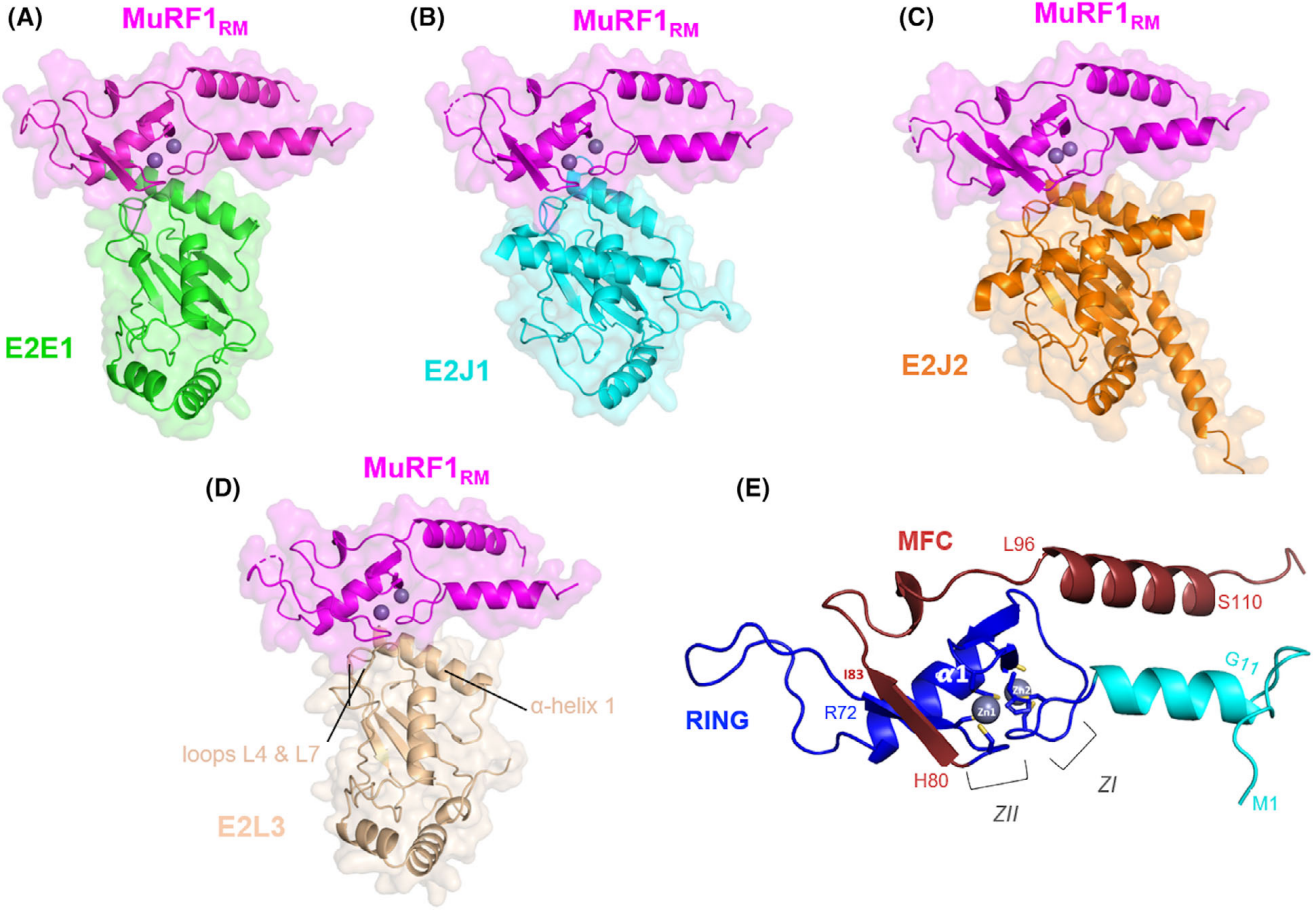
Structure predictions of the different MuRF1‐E2 complexes. AlphaFold 3 (AF3) predicted model of MuRF1_RM_ in pink complexed with (A) E2E1 in green, (B) E2J1 in cyan, (C) E2J2 in orange, or (D) E2L3 in teal. Main chains are displayed in cartoon. Protein surfaces are shown in transparency. Residues in disordered regions with low pLDDT (predicted local distance difference test) values are hidden. The spheres correspond to zinc ions. (E) Close‐up of the structure prediction of the RING domain of the MuRF1_RM_ construct. ZI and ZII, the two zinc‐coordination sites; α1, central α‐helix of the RING domain. Some residues at the boundaries of secondary structures are indicated. Cyan, N‐terminal extension; dark blue; classical RING domain; purple, the MuRF specific domain (MFC).

The predictions made by AF2/AF3 for the E2s corresponded to their known previously published structures. In contrast, the structure of the N‐terminal part of MuRF1, including the RING and MFC domains, has never been experimentally determined. The predictions made by AF2/AF3 for MuRF1_RM_ contained the characteristics of a RING domain (Fig. 2), that is, the presence of two β‐strands (β1, AAs 33–36; and β2, AAs 40–44), and a central α‐helix (referred as α1, AAs 45–55) flanked by two zinc‐fingers corresponding to the E2 interaction site (Z_I_ and Z_II_). AF2/AF3 also predicted the presence of an α‐helix preceding the RING domain (AAs 10–21), and a β‐strand (AAs 72–75) following the α1‐helix. The MFC predictions (AAs 80–115) comprised a β‐strand (AAs 79–83) and an α‐helix (AAs 96–111). The predictions effectively position the RING domain of MuRF1 facing the N terminus α1‐helix and loops 4 and 7 of the E2s (Fig. 2A–D) (for a review on RING E3‐E2 structures, see [25]).

### Structural comparison of the MuRF1‐E2 complexes

**Fig. 3:**
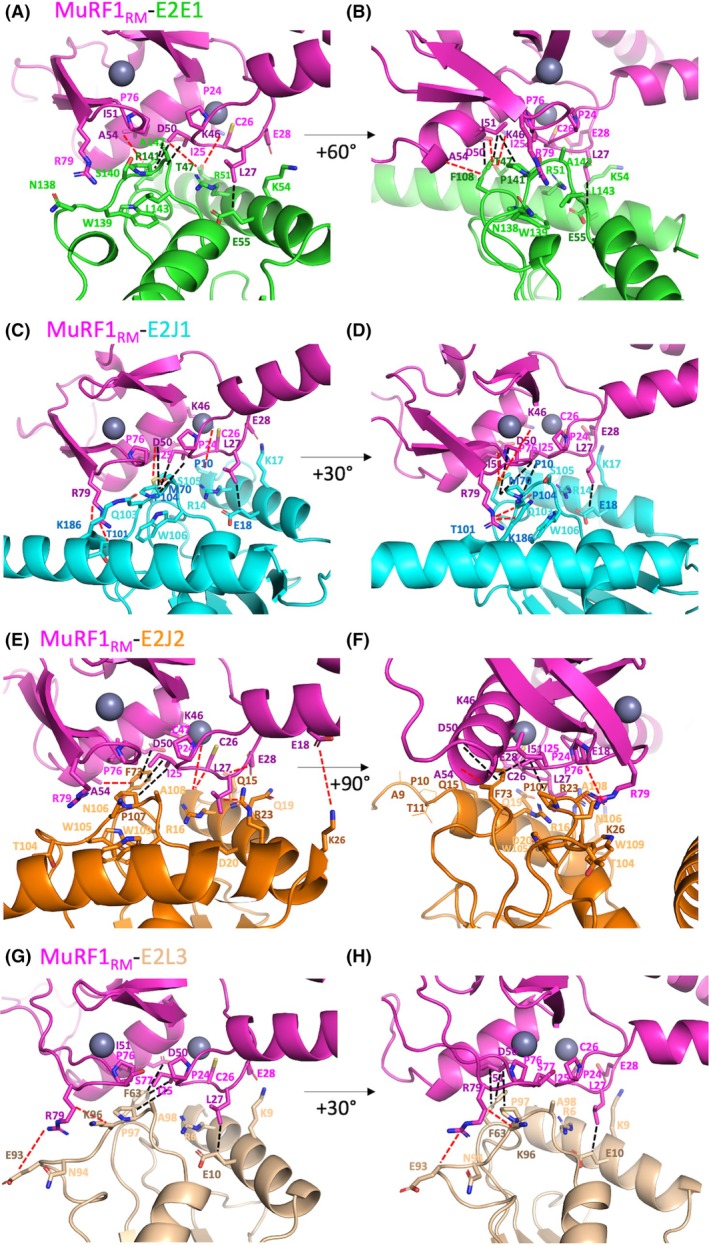
MuRF1‐E2 predicted interfaces. Close‐up view of the protein–protein interfaces to highlight residues at a distance of 4 Å to the partner protein and considered likely to contribute to the interface. Their side chains are displayed as sticks. Distances between residues were calculated with pymol and the images were drawn with pymol. MuRF1_RM_ in pink complexed with (A, B) E2E1 in green, (C, D) E2J1 in cyan, (E, F) E2J2 in orange, or (G, H) E2L3 in teal. Main chains are displayed in cartoon. Residues identified in purple (MuRF1), forest green (E2E1), marine blue (E2J1), brown (E2J2), and chocolate (E2L3) are predicted to be involved in selective contacts and to be specific for the MuRF1‐E2 duos. Residues identified in magenta (MuRF1), bright green (E2E1), cyan (E2J1), light orange (E2J2), and teal (E2L3) are involved in the canonical RING‐type E3‐E2 interaction. Black dashes correspond to contacts present in all MuRF1‐E2 pairs. Red dashes represent selective contacts in a given MuRF1‐E2 pair. Canonical RING‐type E3‐E2 contacts are not shown.

**Fig. 4:**
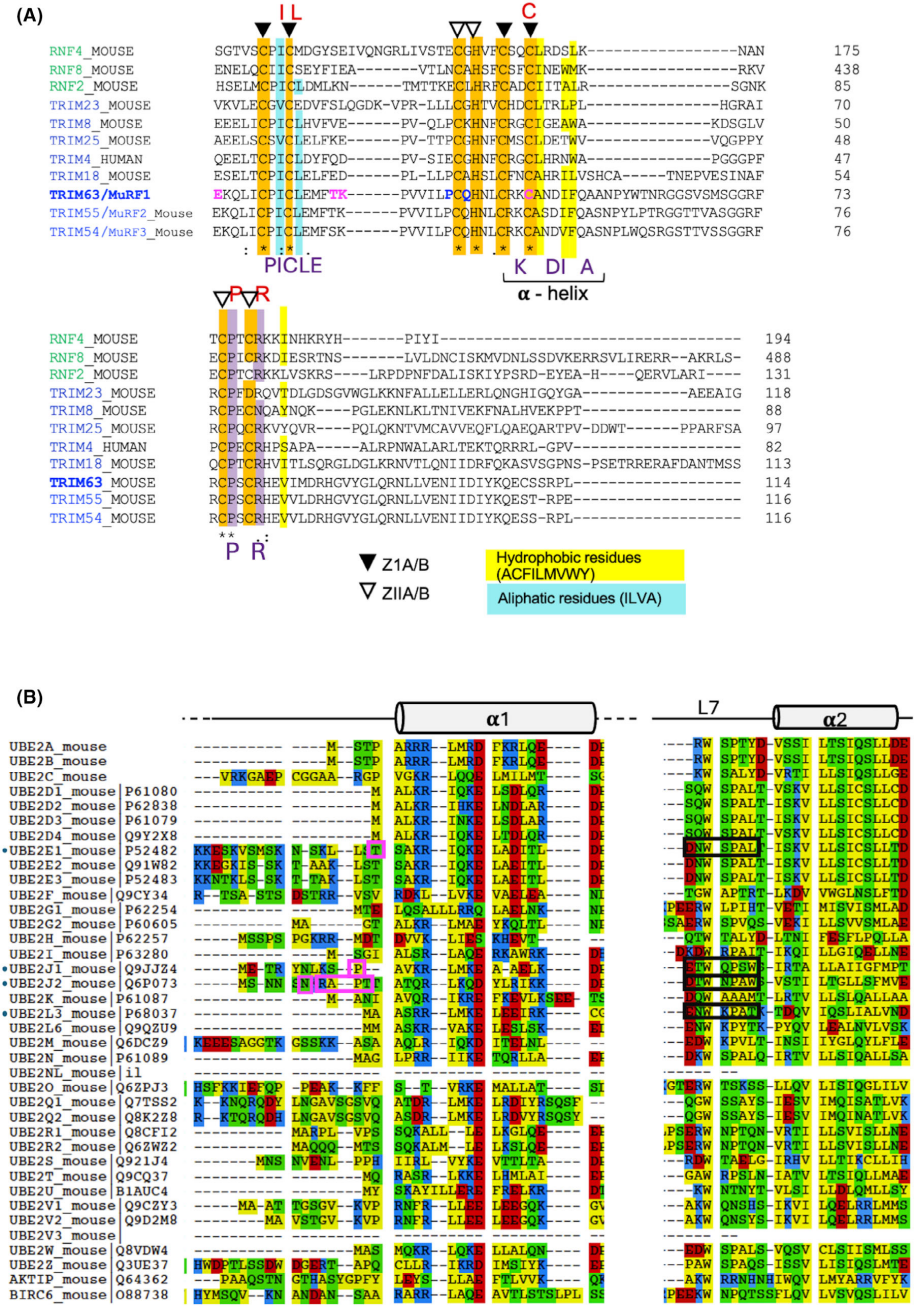
Protein sequence alignments. Sequences were aligned using clustalw. (A) Alignment of RING domains of several RING‐type E3 ligases. The orange shading corresponds to the seven cysteine and the histidine residues that are conserved in the RING domain of RING‐type E3 ligases. These residues coordinate two zinc atoms. ZIA/B, the four cysteines (black triangles) coordinating a zinc atom in the first zinc finger; ZIIA/B, the three cysteines and the histidine (white triangles) coordinating a zinc atom in the second zinc finger. The purple shading corresponds to a proline and an arginine conserved in many RING‐type E3s; the Arg corresponds to the positively charged residue, called “linchpin”, that is involved in the allosteric catalysis of ubiquitination by the RING‐type E3 ligases. The yellow and cyan shading correspond to conserved hydrophobic and aliphatic residues, respectively. The red letters correspond to residues involved in the canonical RING‐type E3‐E2 interactions. The purple residues, below the alignment, correspond to the amino acids involved in MuRF1 interactions with all its E2s partners. Pink residues (E18, T31, and K32) correspond to MuRF1 amino acids specifically involved in the interaction with E2J2. Blue residues (P38 and Q40) correspond to MuRF1 amino acids specifically involved in the interaction with E2J1. (B) Alignment of portions of ubiquitin‐conjugating catalytic (UBC) domain of UBE2 enzymes. N‐terminal part of the UBC domains comprising the α‐helix 1 (α1) and part of the UBC domain including the L7 loop. Red, acidic amino acid (AA); blue, basic AA; yellow, hydrophobic AA; green, uncharged polar AA. Magenta boxes correspond to AA selectively involved in the interaction with MuRF1. Black boxes correspond to the sequence around the conserved motif “SPA” for the E2 MuRF1 partners.

We identified residues as putative contributors to the MuRF1_RM_‐E2 heterodimerization interface when their distance to the partner protein was ≤ 4 Å (Fig. 3), which suggests that they can bind. In both AF2/AF3 predictions, the E2 interface residues correspond to the α‐helix 1, loop L4, and L7 regions, consistent with previous reports. In a recent review, Gundogdu and Walden [25] highlighted the residues involved in canonical RING‐type E3‐E2 interaction, by analyzing the 20 available RING E3‐E2 complexes (note that these E3‐E2 complexes contained only E2D1, E2D2, or E2N as the E2 enzyme). AF2/AF3 was able to identify these canonical non‐selective interactions for the four MuRF1‐E2 couples. Thus, using E2E1 as a reference for residue positioning, AF2/AF3 have identified (a) the side chains of residues Arg51 and Lys54 of E2 α‐helix 1 projecting onto the first zinc‐coordinating center of MuRF1 RING, namely, Ile25, Cys26, Leu27, and Glu28 of the “PICLE” pentapeptidic motif of MuRF1 (ZIA, PICLE motif starting at position 24 on MuRF1; Fig. 4); (b) A bulky AA from the L4 loop (Phe108 for E2E1, Met710 for E2J1, Phe73 for E2J2, and Phe63 for E2L3) also contacting the first zinc‐coordinating center. (c) We also found the well‐conserved SPA motif (Ser141, Pro142, and Ala143 on E2E1), which projects to the first zinc‐coordinating center of RING and the conserved Pro76 and linchpin arginine Arg79, at the end of the RING domain. Please note that Pro76 is conserved in many RING‐type E3 (Fig. 4). (d) AF2/AF3 also predicted a contact between Arg79, at the end of the RING domain (R79), and a 3‐residue motif (position 139 for E2E1) composed of hydrophobic and polar AA, just before the well‐conserved SPA motif. MuRF1 Arg79 is conserved in many RING‐type E3s (Fig. 4) and corresponds to the positively charged residue, called “linchpin”, that is involved in the allosteric catalysis of ubiquitination by the RING‐type E3 ligases [43]. Overall, this confirms that AF2/AF3 has consistently positioned the RING domain of MuRF1 and its E2 partners, in accordance with the previously published RING‐type E3‐E2 structures.

In addition to these canonical contacts, AF2/AF3 also predicted contacts that are present in all MuRF1‐E2 duos (Fig. 3): (a) a Glu residue (Glu55 on E2E1) pointing to Leu27 in the first zinc‐coordinating center of MuRF1; (b) the bulky and hydrophobic Phe108 pointing to several residues of the central α‐helix of MuRF1 (the lateral chain of Asp50 and Ile51 and the backbone carbonyl of Ala54); (c) the Pro142 also projecting to Ile51 of the central α‐helix of MuRF1; and (d) finally, Ala143 making additional contact with the first zinc‐coordinating center.

More interestingly, AF2/AF3 also predicted contacts that appear to be specific for a MuRF1‐E2 pair (Fig. 3) and that thus may be responsible, at least in part, for the selectivity of the MuRF1‐E2 interactions. The selectivity of MuRF1 against E2E1 is concentrated in the central α‐helix of the RING domain of MuRF1, and more precisely, Lys46, Asp50, and Ala54 (KDA motif) (Figs 3A,B and 4). Indeed, the Thr47 of E2E1 points to Lys46 and Asp50 of MuRF1, and the Phe108 of E2E1 points to the backbone carbonyl of Ala54. Interestingly Thr47 is located upstream of the α1‐helix of the UBC fold, and neutral uncharged residues like Thr are rare at this position.

The MuRF1‐E2J1 duo presented more putative‐specific contacts. The same specific KDA motif within the central α‐helix of MuRF1 contacts different AAs on E2J1, namely, Pro10, positioned upstream of the UBC domain, and Met70, equivalent to Phe108 from E2E1. Additionally, the “linchpin” Arg79 of MuRF1 pointed to Thr101 and Lys186 on E2J1. AF2/AF3 also predicted interactions with residues within or positioned after the E2J1 transmembrane domain, which may represent an *in silico* prediction bias.

Predictions for the MuRF1‐E2J2 complex revealed numerous specific contacts. These specific contacts involved the MuRF1‐specific AAs within the central α‐helix (K46, D50, and A54) pointing to Phe73, and also toward an E2J2‐specific motif, RAPT (starting at position 8), in the N‐terminal part of the protein (Figs 3E,F and 4). The RAPT motif may also establish additional contacts with the PICLE motif of MuRF1. Furthermore, in these predictions, MuRF1‐specific AAs, positioned outside the first zinc coordination center and the central helix of MuRF1, pointed to specific residues of E2J2. Hence, the Glu18 of MuRF1 pointed to Lys26 of E2J2, and Thr31 and Lys32 of MuRF1 pointed to Arg8 and Asn6, respectively.

Regarding the MuRF1‐E2L3 pair, only one selective interaction was predicted by AF2/AF3, namely, an interaction between the Arg79 “linchpin” of MuRF1 and the Glu93 of E2L3 (Figs 3G,H and 4). Compared to known complexes between E2L3 and RING‐type E3 ligases (TRAF6, RBX2, and C‐CBL), AF2/AF3 predicted additional contact points for the MuRF1‐E2L3 pair involving residues common to all E2s, that is, Arg6 and Lys9. AF2/AF3 also predicted other E2L3 residues involved in MuRF1 interaction, with Glu10 pointing to Leu 27 of MuRF1 and Ala98 pointing to Pro24 of MuRF1.

### The hydrogen and ionic components of the interaction are different for MuRF1‐E2 pair

**Fig. 5:**
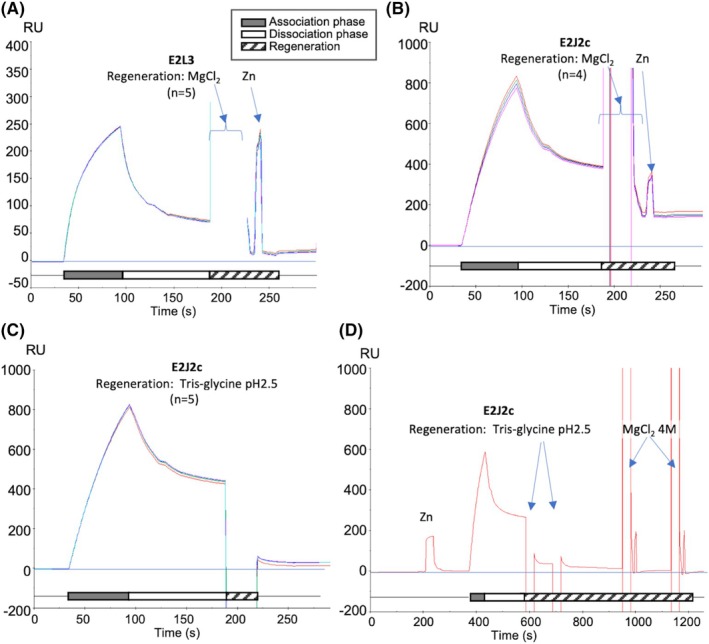
The interaction between MuRF1 and E2L3 is predominantly ionic, while it involves predominantly hydrogen bonds with E2J2c. Surface plasmon resonance (SPR) experiments were performed using a BIAcore T200 instrument. E2L3 and E2J2c were injected (gray bars) at 100 and 500 nm, respectively, in parallel onto the GST and GST‐MuRF1_RM_ surfaces to subtract potential non‐specific interaction with the surface and/or GST. Hatched bar, injection of regeneration solutions (MgCl_2_ or Tris‐glycine pH 2.5). Regeneration of the MuRF1_RM_ surface was completed by a 30‐s pulse of ZnSO_4_. RU, arbitrary response unit. (A) Effect of MgCl_2_ on MuRF1‐E2L3 interaction (*n* = 5). (B) Effect of MgCl_2_ on MuRF1‐E2J2c interaction (*n* = 4). (C) Effect of Tris‐glycine pH 2.5 on MuRF1‐E2J2c interaction (*n* = 5). (D) Complete regeneration of MuRF1_RM_ was achieved by the sequential injections of Tris‐glycine and MgCl_2_ (*n* = 1). *n*, number of replicates.

The nature of the different residues predicted at the MuRF1‐E2 interfaces suggested differences in the driving forces of PPIs and thus the ability of MuRF1 to discriminate between different E2s. In this regard, the two most opposite MuRF1‐E2 pairs were MuRF1‐E2L3 and MuRF1‐E2J2. To verify if this was indeed the case and to have more confidence in the predictions of AlphaFold, we performed complex dissociation experiments, using surface plasmon resonance (SPR) technology. We subjected the MuRF1‐E2L3 and MuRF1‐E2J2 complexes, to different solutions, called “regeneration” solutions to determine which solution would have the greatest effect on their dissociation (Fig. 5). A solution of MgCl_2_ (2 or 4 m) was used to disrupt mainly ionic interactions, and a solution of Tris‐glycine pH 2.5 was used to disrupt hydrogen bonding.

Injection of 2 m MgCl_2_ efficiently disrupted the MuRF1‐E2L3 interaction (Fig. 5A), confirming that this interaction was predominantly ionic. In contrast, injection of MgCl_2_ only partially disrupted the MuRF1‐E2J2c interaction indicating that this interaction was not solely driven by ionic interactions (Fig. 5B). To evaluate the contribution of hydrogen bond at the MuRF1‐E2J2c interface, we then examined the effect of Tris‐glycine solution at pH 2.5 on MuRF1‐E2J2c (Fig. 5C). In this condition, most of the MuRF1‐E2J2c complex was disrupted, confirming that the MuRF1‐E2J2c interaction involves predominantly hydrogen bonds, in contrast to E2L3. The surface was then completely regenerated by the addition of MgCl_2_, reflecting a less important ionic component to the MuRF1‐E2J2c interaction (Fig. 5D). Taken together, these results suggested that, on the whole, the predictions of AF2/AF3 should be correct. They also indicated that the interactions between MuRF1 and its E2 partners do involve different residues that are specific to a single MuRF1‐E2 pair.

### A conserved hydrophobic residue and selective residues govern the interactions of the various MuRF1‐E2 pairs

**Fig. 6:**
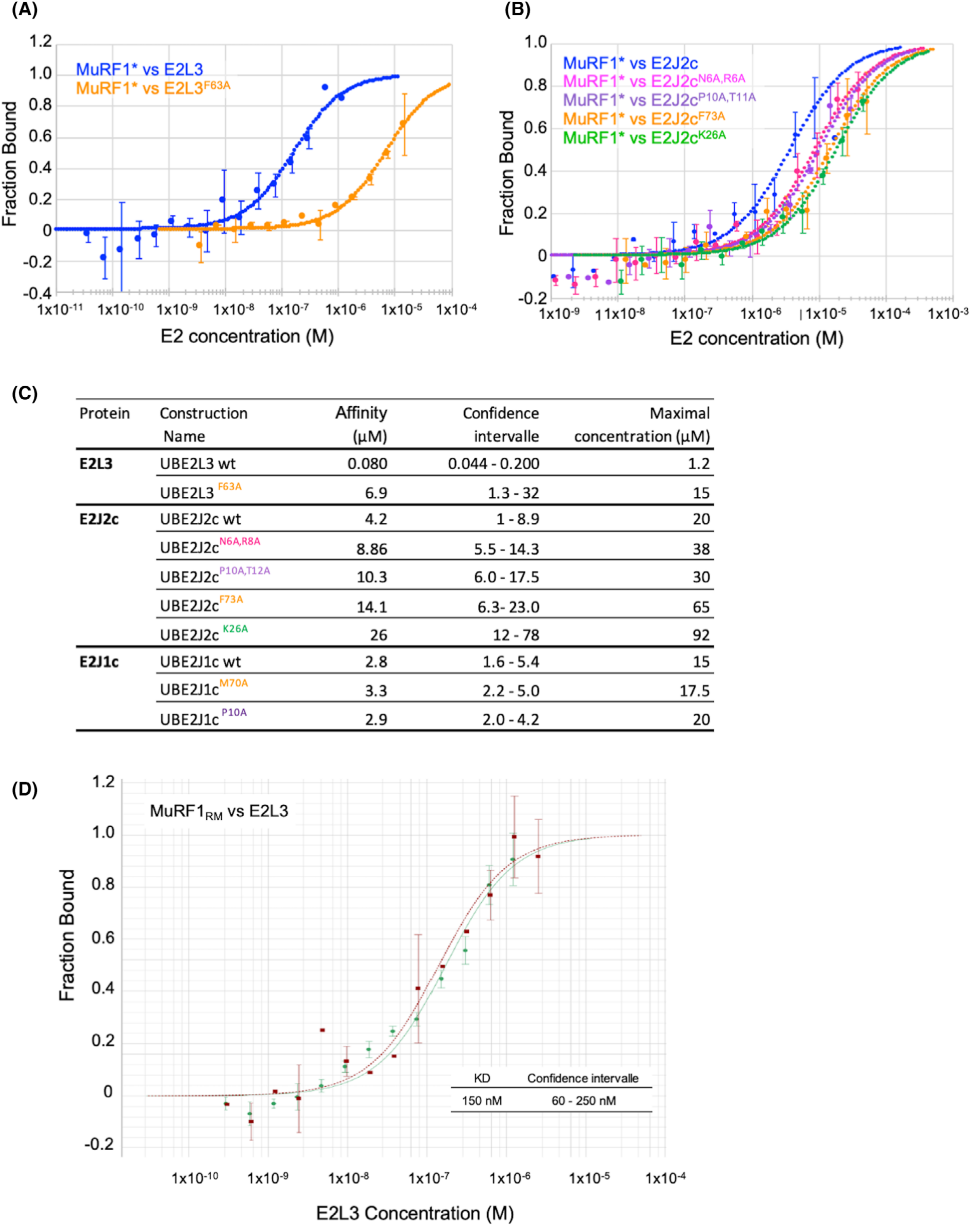
Conserved Phe and selective residues govern the MuRF1‐E2 interactions. Fluorescent MuRF1 was mixed with an E2 protein subjected to a 16‐fold serial dilution. The change in initial MuRF1 fluorescence in the presence of E2 was monitored and plotted against E2 concentration. To facilitate comparison between the different dose–response curves, the change in fluorescence was transformed into “fraction bound”, where a value of 0 (*y*‐axis) corresponds to all free MuRF1 molecules in solution and a value of 1 corresponds to all MuRF1 molecules bound to an E2. (A) Dose–response curves comparison between E2L3 wild‐type and E2L3^F63A^ mutant. Two independent experiments were performed (with three replicates for each experiment) (B) Dose–response curves comparison between E2J2c wild‐type and mutants. Two independent experiments were performed (with three replicates for each experiment) (C) Data set overview. Confidence intervals are expressed in μm. The maximum concentration for each assay is indicated. Note that dose–response curves for E2J1 proteins are not shown because no variation was observed between wild‐type and mutants. (D) Binding affinity of E2L3 for MuRF1‐RM. Fluorescent MuRF1_RM_ was mixed with 16‐fold serial dilution of E2L3. Dose–response curves of two independent replicates (with two and four measures for each replicate, respectively). Error bars represent SD.

Since the AF2/AF3 predictions seemed to be correct, we focused on residues predicted to selectively control the interaction between MuRF1 and a given E2 enzyme. We generated point‐mutant recombinant E2 proteins to experimentally address these predictions, except for E2E1 for which no mutant could be generated. We replaced by an Ala the bulky hydrophobic Phe residue involved in the specific interaction between MuRF1 and all its cognate E2s, namely, Phe73 on E2J2, Phe63 on E2L3, and the equivalent position Met70 on E2J1. For E2J1 and E2J2, we also targeted residues predicted to be involved in the interaction selectivity. For E2J1, we focused only on residues located upstream of the transmembrane domain and therefore replaced Pro10 with an Ala. For E2J2, we mutated the residues Gln6, Arg8, Pro10, Thr11, and Lys26 to an Ala. We compared the affinity of the wild‐type and mutant E2 proteins for MuRF1 using the Monolith technologies (see Materials and methods section) (Fig. 6).

First of all, we measured the affinity of MuRF1 for the wild‐type form of E2L3, E2J1c, and E2J2c (Fig. 6). We found a strong affinity between MuRF1 and E2L3, with an affinity constant estimated around 80 nm, which is consistent with previous measurements [42, 44]. For E2J1c and E2J2c, we measured lower affinities of 2.8 and 4.2 μm, respectively. This was consistent with previous semi‐quantitative results we obtained using the yeast two‐hybrid approach [42]. Surprisingly, neither the M70A nor the P10A point mutation, on E2J1c, induced a significant change in the affinity for MuRF1 (Fig. 6C). In the case of E2L3, the F63A point mutation strongly destabilized the MuRF1‐E2L3 interaction as expected from AF2/AF3 predictions and the SPR results (Fig. 5A). Indeed, the affinity reached 6.9 μm, 86 times lower than with the WT form of E2L3. In the case of E2J2c, the mutation of the bulky F73 residue also led to a decrease in affinity for MuRF1 (Fig. 6B,C), although less pronounced than for E2L3. This result was consistent with the previous SPR results (Fig. 5B–D). Interestingly, the mutation of the residues predicted to be specifically involved in the MuRF1‐E2J2 interaction also led to the destabilization of the pair. Indeed, we measured an affinity of 8.9, 10.3, and 26 μm between MuRF1 and E2J2c^N6A,R8A^, E2J2c^P10A,T11A^, and E2J2c^K26A^, respectively. These results confirmed that most of AF2/AF3 predictions were correct for E2L3 and E2J2c but not for E2J1c. Importantly, this confirmed that only a few residues can govern the selectivity of the MuRF1 interaction toward a specific E2. Note that all these mutants behave comparably to wild‐type proteins during preparation (purity, yield, and concentration).

### Differential affinities of E2 enzymes toward MuRF E3 ligases

**Fig. 7:**
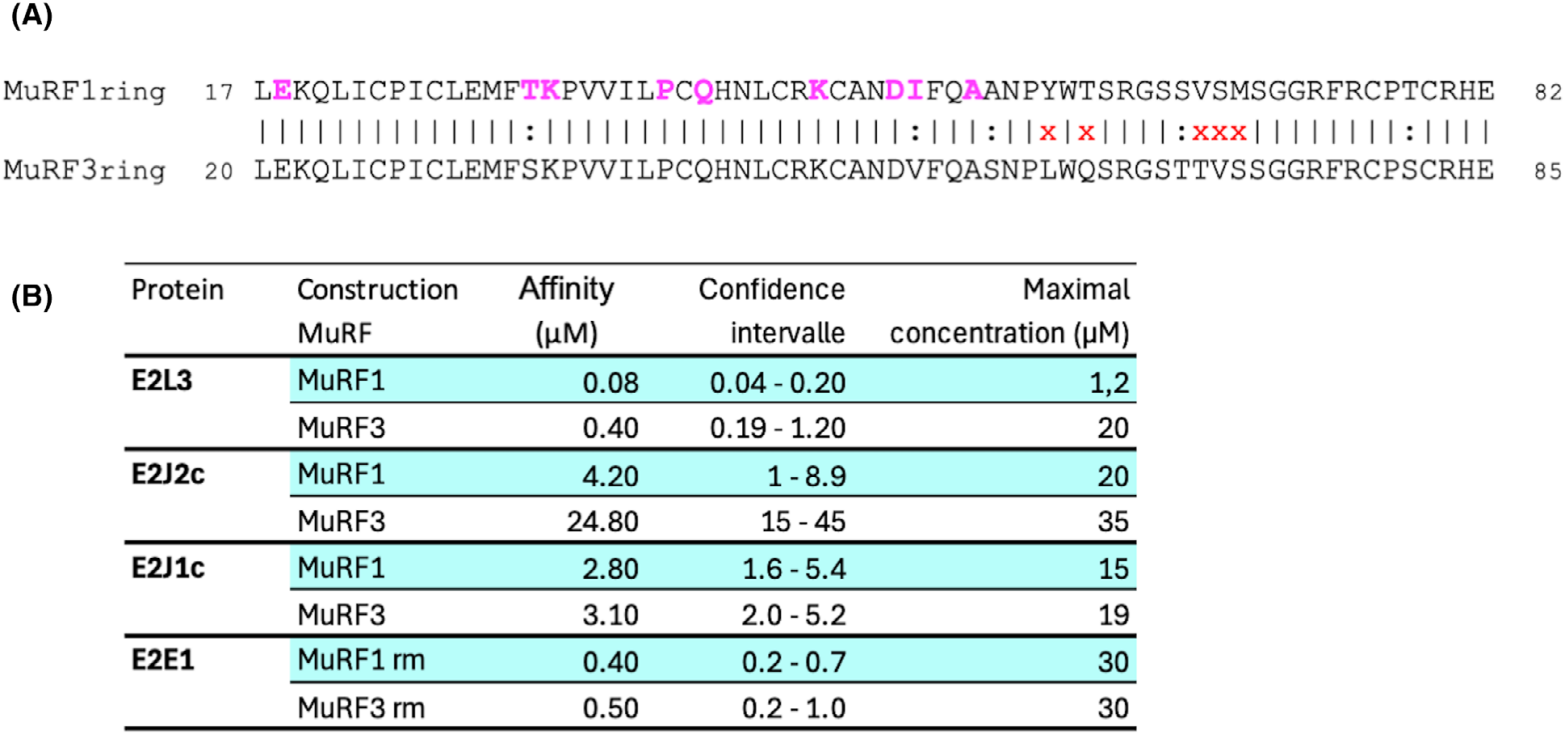
MuRF1 and MuRF3 displayed differences in affinity toward certain E2s. (A) Sequence alignment of the MuRF1 and MuRF3 RING domains. Sequence alignment was performed using the pairwise alignment tool needle (https://www.ebi.ac.uk/Tools/psa/emboss_needle/). A vertical dash indicates positions with a fully conserved residue. A black colon (:) indicates conservation between groups with highly similar properties. A red x indicates non‐conserved residues. The pink residues are involved in specific MuRF1‐E2 interactions. (B) Data set overview of the measured binding constants between MuRF1 or MuRF3 and the E2 proteins, E2E1, E2J1, E2J2, and E2L3. Note that the MuRF_RM_ constructs were used to measure the interaction with E2E1 because the interaction was not stable when using the full length of MuRF proteins. For each interaction, two independent experiments were performed (with three replicates for each experiment).

The RING domains of MuRF1 and MuRF3 are highly conserved (84.6% identity and 92.3% similarity, Fig. 5A). Moreover, the AAs involved in the interaction between MuRF1 and its E2 partners are conserved between both MuRFs (Figs 4A and 7A), suggesting that they can both interact with the same E2s. However, their RING domain contains 5 AAs presenting different properties, out of 65 residues. Given that E2‐E3 PPI selectivity relies on just a few selective residues, we addressed whether MuRF1 and MuRF3 could interact with the same E2, in the same way.

**Fig. 8:**
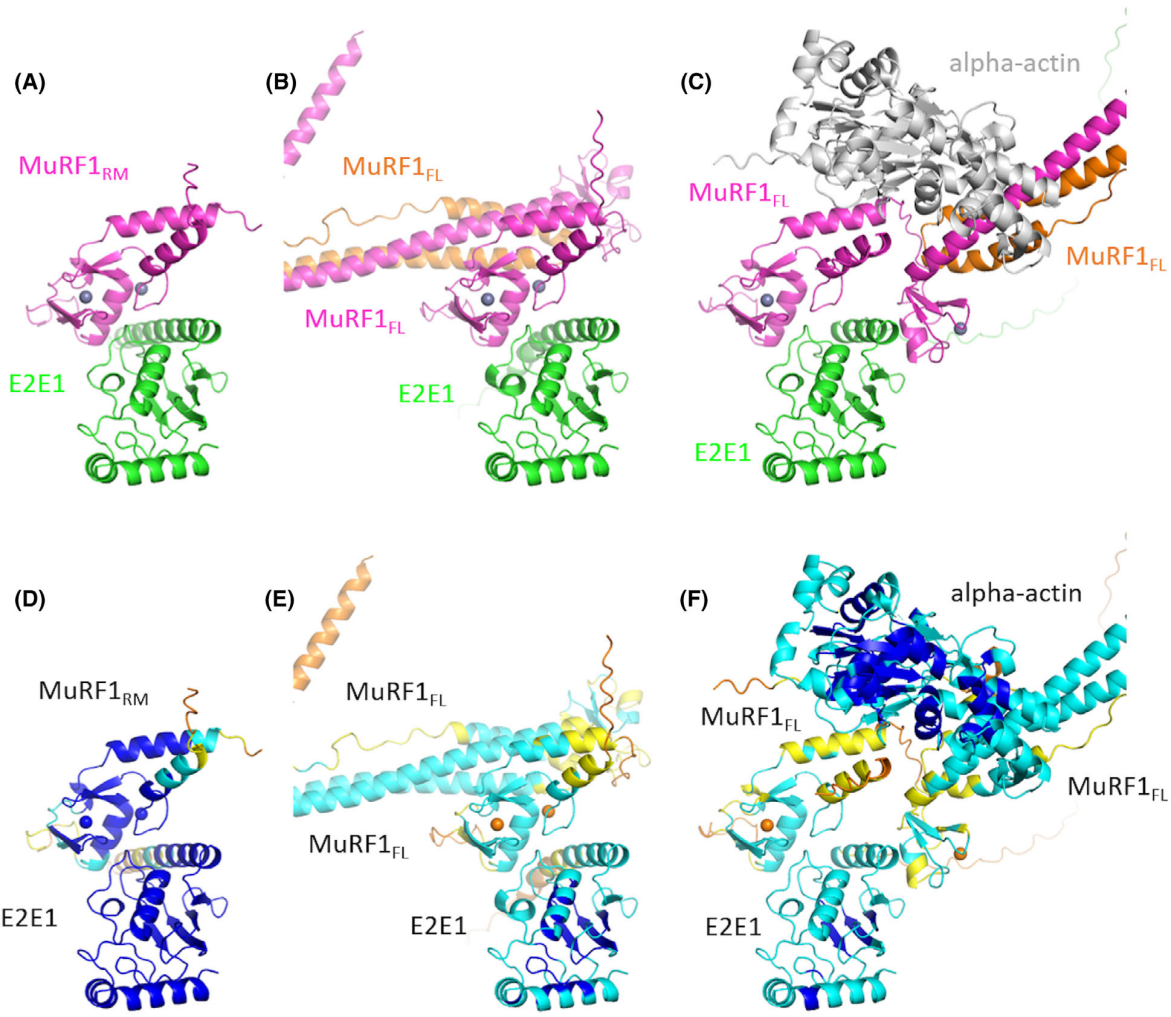
Structure predictions MuRF1‐E2E1 complexed with alpha‐actin. On top, AlphaFold 3 (AF3) predicted models of (A) MuRF1_RM_‐E2E1, (B) homodimeric MuRF1_FL_‐E2E1, and (C) homodimeric MuRF1_FL_‐E2E1 complexed with alpha‐Actin. MuRF1 is colored pink and orange, E2E1 in green, and alpha‐actin in gray. At the bottom, (D–F) are identical projections colored according to AF pLDDT (predicted local distance difference test) score from orange (low pLDDT) to dark blue (high pLDDT). Models are colored according to a metric of per‐residue confidence: The predicted local distance difference test (pLDDT, ranging from 100 to 0), displaying the expected quality of the model. Very high confidence residues are colored in dark blue (pLDDT > 90), high confidence in light blue (90 > pLDDT > 70), low confidence in yellow (70 > pLDDT > 50), and very low confidence in orange (pLDDT < 50). The gray spheres correspond to a pair of zinc added as requested input ions in the AF3 prediction.

MuRF3 was labeled with the fluorochrome NT115 and incubated with increasing concentrations of each E2 (E2E1, E2J1c, E2J2c, or E2L3). Affinity was measured as the change in MuRF3 fluorescence induced by the binding of the E2 enzyme. As for MuRF1, the interaction between the full length of MuRF3 and E2E1 was unstable and was not measurable using the Monolith technology [44]. By contrast, the use of the truncated forms of MuRF1 and MuRF3 containing only the RING and MFC domains (MuRF1_RM_ and MuRF3_RM_) allowed the stabilization of the interaction with E2E1 (Fig. 7B). The affinity measured between MuRF1_RM_ or MuRF3_RM_ and E2E1 was similar, that is, 0.4 and 0.5 μm, respectively. This meant that residues outside the RING domain negatively affected the MuRF‐E2E1 interaction. However, these residues were not predicted by AF2/AF3, when we submitted the full length of MuRF1 and E2E1 (Fig. 8).

MuRF3 also interacted with E2J1c, with almost the same affinity as MuRF1 (0.4 μm). We also measured an interaction between MuRF3 and E2L3 and MuRF3 and EJ2c, but in these cases the affinity (KD) was five to six times weaker than with MuRF1 (Fig. 5B): KD_MuRF3‐E2L3_ = 0.4 μm > KD_MuRF1‐E2L3_ = 0.08 μm and KD_MuRF3‐E2J2c_ = 24.8 μm > KD_MuRF1‐E2J2c_ = 4.2 μm. Collectively, these results indicated that MuRF1 and MuRF3 did interact with the same four E2s, but with differential affinities depending on the E2 involved. This suggested that other residues, than those predicted by AF2/AF3, are involved in the MuRF‐E2 interactions.

## Discussion

The ability of MuRF1 to interact with four different E2s enzymes *in cellulo* probably reflects the need to regulate different physiological functions. Within the cell, MuRF1 must selectively recognize its E2 partners. The identification of the underlying mechanisms responsible for this recognition selectivity is mandatory for selectively drugging discrete functions of MuRF1. To decipher the mechanisms of selective recruitment of E2s by RING‐type E3 ligases, we compared, in this study, the interaction between the RING‐type E3 MuRF1/TRIM63 and four of its E2 partners. We found that the canonical contacts previously described between RING‐type E3s and E2 enzymes are present in the different MuRF1‐E2 duos. In addition, we identified two additional classes of residues involved in the binding selectivity of MuRF1 with its E2 partners. Indeed, we identified (a) residues specifically present on MuRF1 and involved in the interaction with all its cognate E2s and (b) residues responsible for the selective binding of MuRF1 to each E2. These residues are conserved in rodents and humans suggesting an evolutionarily conserved mechanism of interaction selectivity.

A common mechanism has been described that drives the binding of RING‐type E3 ligases and E2‐conjugating enzymes, involving in particular an important hydrophobic component [36, 43, 45, 46]. However, this mechanism is based on the few structures available for an E2 in complex with a RING‐type E3. In addition, most of the studied complexes contained the same E2s, namely, E2L3, E2D1, E2D2, or E2N; E2Ds and E2N being promiscuous E2s interacting with most E3s. The structural predictions we made using AF2/AF3 did indeed find the AAs involved in these canonical E2‐RING‐E3 interactions, both on E2 and on MuRF1. Indeed, for one of these residues, the bulky hydrophobic Phe residue on loop L4 of the E2s, we have shown that an Ala mutation on E2J2 and E2L3 destabilizes MuRF1 binding. It should be noted that this bulky Phe63 has been implicated in the interaction of E2L3 with several E3 ligases [37, 47], confirming a conserved mechanism for a subfamily of E2 rather than a selective mechanism of interaction.

In this work, we showed that MuRF1 exhibits different binding affinities depending on its E2 partners, confirming that other residues are involved in the MuRF1‐E2 interaction. Accordingly, this Phe is present in many E2s, namely, members of the E2E, E2L, E2Q, E2D, E2H, E2J2, E2K, E2W, and E2T families. Since MuRF1 does not interact with many of these E2s *in cellulo* or *in vitro* [42, 48], this confirms that this residue is not sufficient for an E2 to interact with MuRF1.

The presence of this canonic Phe may explain some discrepancies observed in the literature. Notably, *in vitro* ubiquitination has been described by using MuRF1 and E2D2, E2N/V, or E2W [16, 49, 50], but none of these studies has ever detected any affinity between MuRF1 and these E2s. By contrast, we found that E2D2 has no affinity for MuRF1 either *in vitro* or *in cellulo* [42, 48]. This discrepancy can be explained by the high processivity of E2D2 *in vitro* [51] and potentially by a very weak and non‐physiological affinity of MuRF1 for E2D2 due to the presence of the canonical Phe. Indeed, the interaction between the two partners can be artificially enhanced *in vitro*, for example, by using supraphysiological concentrations of E2s and E3. As an example, this is the case of the E3 ligase E6AP that interacts *in vitro* with E2L3 and E2D2 but with very different binding affinities of 5 and 160 μm, respectively [47]. Whether an affinity of 160 μm has any physiological significance is a legitimate question. Very recently, E2W has been shown to work with MuRF1 *in vitro* [50] but the affinity of E2W toward MuRF1 was not reported and the canonical Phe on loop 4 might have been sufficient to generate Ub chains in presence of MuRF1.

We further confirmed the involvement of other residues in the MuRF1‐E2 PPI and we showed that the driving force of MuRF1‐E2 interactions differs between couples. Indeed, using an SPR approach, we found that the interface between MuRF1 and E2L3 was primarily driven by ionic bonds, whereas the interface between MuRF1 and E2J2 was more likely to be driven by hydrogen bonds. This is in contrast to the dogma of a well‐conserved hydrophobic interface, often suggested for the RING‐type E3‐E2 protein–protein interfaces [29, 36, 43, 45, 46, 52]. We further confirmed these results by using point mutations of the AAs predicted to be involved in the selective E2J2c‐MuRF1 interaction, which resulted in a significant decrease in binding affinity. Please, note that the involvement of electrostatic rather than hydrophobic contacts has already been reported for the TRIM21‐E2N pair [33]. This was also demonstrated for the FANCL‐E2T pair, where the selectivity of the interaction was due to additional specific ionic and hydrogen bonds in addition to the hydrophobic components [35]. Combined with the literature, our data show that canonical hydrophobic interactions as well as specific contacts (e.g., electrostatic and hydrogen bonds) are crucial for the interaction selectivity between an E2 and a RING‐type E3. In this work, we provide the molecular basis for the mechanisms that allow MuRF1 to select different E2 enzymes to ubiquitinate specific substrates.

Interestingly, in MuRF1‐E2E1 and unlike the other MuRF1‐E2 pairs, it appears that residues outside the RING domain also contribute to the interaction. Indeed, the interaction between full‐length MuRF1 and E2E1 was not stable *in vitro* and could not be measured using either the Monolith (this study) or the SPR [42]. However, deletion of the C‐terminal part of MuRF1 after the MFC domain (residue 115) stabilized the MuRF1‐E2E1 PPI (from an unstable interaction to a binding affinity of 0.4 μm). This suggests that a domain located C terminally to the MFC domain negatively controls the binding of E2E1. Such an inhibitory mechanism has never been described. In contrast, some E3 ligases have been described to possess a domain‐specific E2‐binding helix distal to their RING domain. This helix, by interacting with the backside of the UBC domain, controls the type of ubiquitination or increases the binding affinity between the E2 and the E3, thereby stimulating the polyubiquitination reaction. This is the case for the GP78‐E2G2, RAD18‐E2B, and RNF25‐E2D2 duos [53, 54, 55]. AF2/AF3 predicted no contact between MuRF1 and the E2E1 backside. In the case of MuRF1‐E2E1, the distant inhibitory domain could be pushed away by the binding of the substrate to MuRF1. Indeed, we have previously shown that the presence of myofibrillar alpha‐actin, a known substrate of MuRF1, strongly stabilizes the MuRF1‐E2E1 interaction (affinity of ~ 70 nm) [44], indicating that the E2 binding on RING‐E3 may also be substrate dependent. We included dimeric full‐length MuRF1 (MuRF1_FL_) in the AF3 prediction (Fig. 8B,E) and alpha‐actin as a substrate as part of the complex (Fig. 8C,F). However, this did not help to conclusively clarify our hypothesis. We found the same contact points using a homodimer of MuRF1_FL_ instead of MuRF1_RM_, the difference being a lower confidence of the model (compare Fig. 8E and 8D). The addition of alpha‐actin led to a worse prediction by AF3, with important local modifications of the MuRF1 conformation (Fig. 8F), although mostly in the periphery of the RM domain and with minor implications of E2E1. Further experiments would be required to decipher the precise mechanisms of MuRF1‐E2E1 interaction, and an experimental high‐resolution structure would be useful in this case.

Surprisingly, no additional selective interaction was predicted by AF2/AF3 for the MuRF1_RM_‐E2L3 pair, despite this pair having the highest affinity. Using the full‐length form of MuRF1 for AF2/AF3 predictions also failed to reveal additional contacts between MuRF1 and E2L3. These additional contacts likely involve residues from the RING or MFC domains of MuRF1, since the affinity for E2L3 measured using the full‐length form of MuRF1 (MuRF1_FL_) or MuRF1_RM_ was similar (Fig. 6). AF2/AF3 also predicted contact points between MuRF1 and E2J1 that were not experimentally confirmed. This study shows that AF2/AF3 artificial intelligence was a useful tool to predict some, but not all, AAs responsible for the binding selectivity between an E2 enzyme and an E3 ligase. Only an experimental determination of the structure of the MuRF1‐E2 duos, for example, by NMR or mass spectrometry, could help to determine all the crucial contact points.

MuRF1 belongs to a small family consisting of three members in mammals, namely, MuRF1, MuRF2, and MuRF3. MuRF1 and MuRF3 share 70% homology and are expressed in adult muscles [56]. Residues involved in MuRF1‐E2 interactions are also conserved in the RING domain of MuRF3. This high degree of conservation suggests that both E3 ligases may interact with the same E2s similarly. We showed that MuRF1 and MuRF3 interacted with the same four E2s (E2E1, E2J1, E2J2, and E2L3), in accordance with previous reports indicating that, MuRF1, MuRF2, and MuRF3 can ubiquitinate identical substrates *in vitro* [16, 50]. However, we have shown in the present study that the affinities between the MuRFs and each E2 can vary by different orders of magnitude, with MuRF1 exhibiting a better affinity for E2L3 and E2J2 than MuRF3. This suggests that *in vivo* the MuRFs may interact preferentially with different E2 enzymes, each MuRF1‐E2 pairs targeting different substrates. This difference in binding affinity further strengthens the hypothesis that other residues not predicted by AF2/AF3 are involved in the MuRF‐E2 interactions, which may involve only a few residues as previously described in the literature [36, 37, 47]. Between MuRF1 and MuRF3, there are five non‐conserved residues and five different residues with similar properties. It is noteworthy that, despite the structural conservation of the UBC domain in E2s, loops L4 and L7 exhibit high variability in sequence, length, and conformational freedom [24], and the central α‐helix 1 of the E3 RING domain also varies widely in length [25], which provides different possibilities to achieve selectivity.

The limitations of this study are that the results were obtained using *in silico* and *in vitro* approaches. It will then be necessary to confirm these results *in cellulo*. Indeed, if we design a drug inhibiting MuRF1‐E2 interaction, this should preserve MuRF1 targets and myotube atrophy under catabolic conditions. However, this work is a good starting point for the understanding of the mechanisms underlying the selective interaction between MuRF1 and its E2 partners.

The results of the present study support the idea that the more than 600 RING‐type E3s, despite their structural similarity, have evolved to discriminate among 38 structurally related E2s. These evolutions involve modifications within and outside the RING domain. It is likely that multiple mechanisms will be discovered that govern the selective interaction of each E2‐E3 pair. Knowing the selective aspects of the E2‐E3 interaction, the potential for creating therapeutics by integrating structure‐based drug design becomes increasingly promising. For each pair of interest, it will then be necessary to determine the residues and mechanisms that are responsible for the selectivity of the interaction. Such a strategy could be used to selectively regulate MuRF1 function leading to muscle atrophy.

## Materials and methods

### Structure predictions

MuRF1_RM_ corresponds to a truncated form of MuRF1 comprising amino acids 1–115. The candidate domains sequence of MuRF1_RM_ and UBE2E1, UBE2J1, UBE2J2, or UBE2L3 (Table 1) were submitted to AF2 or AF3 folding and hetero‐dimerization prediction [39, 41, 57] in the ColabFold or https://alphafoldserver.com standard setups, respectively [40]. For AF3, a pair of zinc was modeled. Figures representing structural models were drawn with pymol (Schrodinger Inc., New York, NY, USA).

**Table 1:** Protein sequences used in this study.

Organism	Protein	Sequence
*Rattus norvegicus*	MuRF1_RM_	MDYKSGLIPDGNAMENLEKQLICPICLEMFTKPVVILPCQHNLCRKCANDIFQAANPYWTNRGGSVSMSGGRFRCPSCRHEVIMDRHGVYGLQRNLLVENIIDIYKQECSSRPLQ
*Rattus norvegicus*	MuRF1_FL_ TRIM63 Q91Z63	MDYKSGLIPDGNAMENLEKQLICPICLEMFTKPVVILPCQHNLCRKCANDIFQAANPYWTNRGGSVSMSGGRFRCPSCRHEVIMDRHGVYGLQRNLLVENIIDIYKQECSSRPLQKGSHPMCKEHEDEKINIYCLTCEVPTCSLCKVFGAHQACEVAPLQSIFQGQKTELSNCISMLVAGNDRVQTIISQLEDSCRVTKENSHQVKEELSHKFDALYAILDEKKSELLQRITQEQEEKLDFIEALILQYREQLEKSTKLVETAIQSLDEPGGATFLLSAKPLIKSIVEASKGCQLGKTEQGFENMDYFTLNLEHIAEALRAIDFGTDEEEEFTEEEEEEDQEEGVSTEGHQ
*Mus musculus*	UBE2E1 P52482	MSDDDSRASTSSSSSSSSNQQTEKEGSTPKKKESKVSMSKNSKLLSTSAKRIQKELADITLDPPPNCSAGPKGDNIYEWRSTILGPPGSVYEGGVFFLDITFTPEYPFKPPKVTFRTRIYHCNINSQGVICLDILKDNWSPALTISKVLLSICSLLTDCNPADPLVGSIATQYMTNRAEHDRMARQWTKRYAT
*Mus musculus*	UBE2J1 Q9JJZ4	METRYNLKSPAVKRLMKEAAELKDPTDHYHAQPLEDNLFEWHFTVRGPPDSDFDGGVYHGRIVLPPEYPMKPPSIILLTANGRFEVGKKICLSISGHHPETWQPSWSIRTALLAIIGFMPTKGEGAIGSLDYTPEERRALAKKSQDFCCEGCGSAMKDVLLPLKSGSGSSQADQEAKELARQISFKAEVNSSGKTIAESDLNQCFSLNDSQDDLPTTFQGATASTSYGAQNPSGAPLPQPTQPAPKNTSMSPRQRRAQQQSQRRPSTSPDVLQGQPPRAHHTEHGGSAMLIIILTLALAALIFRRIYLANEYIFDFEL
*Mus musculus*	UBE2J2 Q6P073	MSNNSNKRAPTTATQRLKQDYLRIKKDPVPYICAEPLPSNILEWHYVVRGPEMTPYEGGYYHGKLIFPREFPFKPPSIYMITPNGRFKCNTRLCLSITDFHPDTWNPAWSVSTILTGLLSFMVEKGPTLGSIETSDFTKKQLAAQSLVFNLKDKVFCELFPEVVEEIKQKQKAQDELSNRPQNLPLPDVVPDGELHRGQHGIQLLNGHAPAAGPNLAGLPQANRHHGLLGGALANLFVIVGFAAFAYTVKYVLRSIAQE
*Mus musculus*	UBE2L3 P68037	MAASRRLMKELEEIRKCGMKNFRNIQVDEANLLTWQGLIVPDNPPYDKGAFRIEINFPAEYPFKPPKITFKTKIYHPNIDEKGQVCLPVISAENWKPATKTDQVIQSLIALVNDPQPEHPLRADLAEEYSKDRKKFCKNAEEFTKKYGEKRPVD

### Constructs

Using Superscript II and Platinum Pfx DNA polymerase (Invitrogen, Thermo Fisher Scientific, Courtaboeuf, France), we amplified by RT‐PCR the full‐length UBE2E1 and UBE2L3, and the cytosolic part of UBE2J1 (E2J1c) and UBE2J2 (E2J2c) from murine C2C12 skeletal muscle cells. cDNAs coding for E2 proteins, MuRF1, MuRF3, and their truncated form (MuRF1_RM_ and MuRF3_RM_; aa 1–115) were cloned in the expression vectors pET28a (Novagen, Merck, Fontenay‐sous‐Bois, France) and pGEX6P3, respectively, for the production of recombinant proteins in *Escherichia coli*. Mutated forms of E2 have been synthesized by GeneCust (Boynes, France).

### Protein expression and purification

GST‐MuRF1, GST‐MuRF1_RM_, GST‐MuRF3, and GST‐MuRF3_RM_ were expressed and purified using Sepharose 4B affinity matrix (Cytiva, Saint‐Germain‐en‐Laye, France) as previously described for MuRF1 in [2]. UBE2 proteins were expressed in *E. coli* (*E. coli* Rosetta (DE3); Novagen) as His‐tag fusion proteins. They were purified on Ni‐NTA agarose matrix (Qiagen, Courtaboeuf, France) according to supplier instructions. The protein buffer was then exchanged by dialysis (50 mm HEPES buffer pH 7.4, 150 mm NaCl, 0.005% v/v Tween20, and 0.1% dextran) for subsequent affinity measurement experiments. Note that only the cytoplasmic parts of E2J2 and E2J1, called E2J2c and E2J1c, respectively, were used in the *in vitro* experiments.

### Crystallization

For crystallization, GST‐MuRF1_RM_ was purified by affinity chromatography, and the N‐terminal GST‐tag of GST‐MuRF1_RM_ was cleaved by incubation with the Prescission Protease (Cytiva), according to the supplier recommendations. After affinity purification, MuRF1_RM_ and E2E1 or E2L3, E2J2c, or E2J1c, were mixed at a 2 : 1 ratio and submitted to size exclusion chromatography (Superdex 200 26/600) to purify the complex. Fractions corresponding to the size of the dimer were analyzed by SDS/PAGE to confirm the presence of both MuRF1 and E2. Purified MuRF1_RM_‐E2 complexes were submitted to extensive sparse‐matrix vapor diffusion crystallization screens (384 JCSG conditions) in 100–200 nL sitting drops at 20 °C.

### SPR experiments

SPR experiments were performed with a BIAcore T200 instrument (Cytiva) at 25 °C. GST‐MuRF1_RM_ and GST were covalently immobilized on a CM5 sensor chip by standard amine coupling. Interaction measurements were performed in running buffer (10 mm HEPES pH 7.4, 150 mm NaCl, and 0.05% (v/v) surfactant P20) at a flow rate of 30 μL·min^−1^. E2L3 and E2J2c were injected at 100 and 500 nm, respectively, in parallel onto the GST and GST‐MuRF1_RM_ surfaces, during 60 s. One hundred seconds after the end of E2 injection, the regeneration solutions (MgCl_2_ or Tris‐glycine pH 2.5) were applied for 30 or 60 s. A solution of MgCl_2_ (2 or 4 m) was used to disrupt mainly ionic interactions, and a solution of Tris‐glycine pH 2.5 was used to disrupt hydrogen bonding. After the disruption of the MuRF1_RM_‐E2 interaction, regeneration of the MuRF1_RM_ surface was completed by a 30 s pulse of 200 μm ZnSO_4_. The GST surface was used to subtract the background from the signal obtained on the GST‐MuRF1_RM_ surface.

### Binding constant determination

Binding constants were determined by the measure of change in the initial fluorescence of a fluorescent MuRF1 E3 ligase in the presence of the ligand (the wild‐type or mutated forms of UBE2E1, UBE2J1c, UBE2J2c, and UBE2L3), using MonolithX (NanoTemper Technologies, Munich, Germany). GST‐MuRF1, GST‐MuRF3, or GST‐MuRF1_RM_ were covalently labeled with red fluorescent dye NT‐647 (excitation 650 nm; emission 670 nm), by amine coupling, using the Protein Labeling Kit REDNHS (Nano‐Temper Technologies). Labeled MuRF1 concentration was kept constant at 10 nm, while the concentration of the ligand varied. A 16‐sample serial dilution of the ligands was made. Proteins were loaded into premium treated capillaries for measurements using 100% MST power at 25 °C. Data from initial fluorescence were evaluated to derive a binding affinity. An affinity measurement was considered reliable when the signal‐to‐noise ratio was ≥ 10. At least two independent replicates were performed, and within a given replicate, measures were repeated three times. Data analyses were performed using the mo.affinity Analysis software (NanoTemper Technologies).

## Conflict of interest

The authors declare no conflict of interest.

## Author contributions

JH and CP contributed to conception and design of the work; AC, M. Malige, M. Macheton, CP, and JH contributed to data acquisition; JH and CP contributed to data analysis; LC, EL, PF, DT, JH, and CP contributed to interpretation of data; JH and CP contributed to drafting of the manuscript; AC, M. Malige, M. Macheton, LC, EL, PF, DT, JH, and CP contributed to critical review and editing. All the authors approved the published version of the manuscript.

### Peer review

The peer review history for this article is available at https://www.webofscience.com/api/gateway/wos/peer‐review/10.1111/febs.70017.

## Data Availability

All data that support the findings of this study are reported in the text and figures and are available from the corresponding authors cecile.polge@inrae.fr and julien.henri@sorbonne-universite.fr upon reasonable request. Protein sequence data that support the findings of this study are openly available in UniProt at: https://www.uniprot.org/uniprotkb/Q91Z63/entry, primary accession number [Q91Z63], for MuRF1/TRIM63; https://www.uniprot.org/uniprotkb/P52482/entry, primary accession number [P52482], for UBE2E1; https://www.uniprot.org/uniprotkb/Q9JJZ4/entry primary accession number [Q9JJZ4], for UBE2J1; https://www.uniprot.org/uniprotkb/Q6P073/entry, primary accession number [Q6P073], for UBE2J2; https://www.uniprot.org/uniprotkb/P68037/entry, primary accession number [P68037], for UBE2L3.
